# Non-Isothermal Crystallization Kinetics and Activation Energy for Crystal Growth of Polyamide 66/Short Glass Fiber/Carbon Black Composites

**DOI:** 10.3390/ma16227073

**Published:** 2023-11-07

**Authors:** Yasser Boucenna, Abdelheq Layachi, Abdelhakim Cherfia, Fouad Laoutid, Hamid Satha

**Affiliations:** 1 Mechanics Laboratory, Frères Mentouri University Constantine 1, Constantine 25000, Algeria; boucennayasser2525@hotmail.com (Y.B.); cherfia.abdelhakim@umc.edu.dz (A.C.); 2 Institut des Sciences et des Techniques Appliquées (ISTA), Frères Mentouri University Constantine 1, Constantine 25000, Algeria; 3 Laboratory of Silicates, Polymers and Nanocomposites, University 8 Mai 1945, Guelma 24000, Algeria; satha.hamid@univ-guelma.dz; 4 Laboratory of Polymeric & Composite Materials, Materia Nova Research Center, 3 Avenue Nicolas Copernic, B-7000 Mons, Belgium

**Keywords:** PA66, glass fiber, carbon black, DSC, crystallization, activation energies

## Abstract

This study presents the effect of the addition of 0.4 wt.% carbon black (CB) to polyamide 66 (PA66) containing 30 wt.% short glass fibers (GFs) on the behavior of composite thermal crystallization. Composites were studied by differential scanning calorimetry analysis (DSC) at different cooling rates using wide-angle X-ray scattering (WAXS) and scanning electron microscopy (SEM). This thermal crystallization study highlights the nucleation effect of GFs that promote PA66 crystallization by significantly increasing crystallization kinetics and rates. The activation energies (Eas) calculated by model-free (FWO; KAS) and model-fitting (Kissinger method and C–R method) approaches showed that the combination of both GF and CB decreases the activation energy with respect to neat PA66, meaning that the presence of both additives facilitates crystallization. The Coats–Redfern and Criado methods showed that the crystallization of neat PA66 and related composites follows the second-order reaction, i.e., the decelerated reaction, evidencing compatibility between GFs and the matrix.

## 1. Introduction

Polyamides are commonly used in various applications due to their high strength and durability. Polyamide 66 (PA66) is a semicrystalline thermoplastic with excellent thermal and physical properties [1] and high mechanical properties (rigidity, wear, and creep resistance) and boasts exceptional short-term thermal endurance due to its high melting point of 260 °C [2,3].

Industrial polymer-based composites use mineral fillers and fibers as reinforcing agents [4,5,6]. These agents enhance the polymer’s mechanical properties while also decreasing production expenses [3]. When polyamide 66 is reinforced with glass fiber (PA66/GF) and carbon black (PA66/CB), the resulting composite material has excellent mechanical properties. Concurrently, it is commonly understood that fibers can significantly enhance the crystallization process of various thermoplastic polymers with semi-crystalline properties [7,8,9,10,11,12,13,14,15,16,17,18,19]. Glass fibers (GFs) are a popular reinforcement material for composites because of their high strength-to-weight ratio and resistance to corrosion. When added to PA66, glass fibers can significantly improve the composite’s strength and stiffness and increase the elastic modulus above the glass transition temperature of semi-crystalline polyamide [2,8,20]. Carbon black (CB) is also frequently used to enhance the thermal and mechanical properties of the mechanical parts of PA66, such as resistance to wear caused by sliding (abrasion), electrical conductivity, and thermal stability [21]. Like GF, carbon black modifies the crystallization behavior of semi-crystalline polymers [22,23].

The combination of glass fibers and carbon black with PA66 creates a composite material that exhibits superior properties. These properties include high impact, abrasion, and fatigue resistance. This makes it ideal for use in various applications, such as automotive parts, aircraft components, and sporting equipment [1,24].

The crystallization kinetics of semi-crystalline polymers, which have significant influences on the crystal structures, morphologies, and polymer properties, is a critical parameter for polymer processing [25]. The study of non-isothermal crystallization kinetics is very relevant to understanding the properties of composite materials [26]. PA66 crystallization typically occurs during cooling from the melt state. During the cooling process, the polymer chains arrange themselves in a highly ordered manner, forming crystals. Crystals grow and interlock, leading to the formation of a solid material with a highly ordered structure [27].

Polyamide crystallization is influenced by several factors such as temperature, cooling rate, and additives or fillers. In addition, it is influenced by the polymerization degree. It typically occurs during the solidification process of the polymer, either during molding or cooling of the melted polymer [19]. The effectiveness of composite materials utilizing a semi-crystalline polymer matrix relies not only on the proportion of the two base components but also on the ratio of crystallinity and the crystal structure of the matrix [1,2]. Globally, the crystallization process plays a crucial role that affects the final properties of polyamide. Its control is of high importance in optimizing the performance of this technical polymer in various applications.

In recent years, most researchers have focused on studying the non-isothermal crystallization kinetics of polymers and the way in which they can be affected by reinforcing fillers [1,2,3,4,5,6,28,29]. For example, Layachi et al. [1] studied the non-isothermal crystallization kinetics of 15 wt.% glass fiber or 0.4 wt.% carbon black reinforced PA66 at different cooling rates. The results show that the PA66 crystallization rate is increased in the presence of these additives. What is more, Mo’s method can provide a good explanation of the non-isothermal crystallization of neat PA66 and PA66 composites [1,2]. Arroyo et al. [28] studied the effect of short GFs on the isothermal crystallization of a polypropylene matrix. They determined that the presence of a GF reinforcing agent has a notable impact on the nucleation of the polymer. In addition, the incorporation of GFs can expedite the crystallization process, resulting in a shorter crystallization time.

When reinforcing fillers and additives are added to semi-crystalline polymeric matrices, the crystallization capabilities of the polymer matrix are modified [1,2]. This includes changes in crystallization kinetics, crystal perfection, and crystallinity index [29]. Studies have shown that solid particles in such materials often act as nucleating agents during crystallization. As a result, this is likely to affect the mechanical properties of composites. Frihi et al. [29] studied the influences of glass fiber contents on PA66 composites’ crystallization properties. They determined that the PA66/GF crystallinity ratio is significantly influenced by GF composition and processing conditions.

In the process of thermoplastic composite development, a semi-crystalline structure is created alongside interfacial bonds [2,29]. Understanding and managing the role of the reinforcing fibers in matrix crystallization is crucial. The polymer crystalline morphology, which is influenced by nucleation rates and crystal growth, is a crucial factor that affects the mechanical and physical properties of the final product. Therefore, gaining a better understanding and control of this phenomenon is of substantial interest [30,31]. Makhlouf et al. [2] highlighted a reduction in the nucleation effect for composites containing 30 wt.% GF. This was attributed to the fact that the PA66 matrix approaches its saturation point at approximately 30 wt.% GF. The observation of a nucleation optimum at 30% can be attributed to the increase in the crowding effect of glass fibers that gradually impinge into each other and mutually cover each other with increasing contents so that only a part of their surface remains fully active for crystal nucleation. In parallel, the observation from a recent structural study [29] that the PA66 matrix crystallinity gradually saturates at a glass fiber content of 30% is most probably because the increasing number of glass fibers is able to compensate—to some extent—the reduced nucleation activity of the glass fibers. Then, the overall PA66 crystallinity is influenced by the reduction of glass fiber nucleation activity at a GF content higher than 30 wt.%. This is in conjunction with the intrinsic crystallization capability of PA66 upon quiescent conditions. Additionally, the thermal stability of PA66/GF composites surpasses that of neat PA66. This is because of the bonding that occurs between the PA66 matrix and the GF.

The non-isothermal crystallization kinetics and reaction mechanisms of PA66 and its composites need to be understood to better control thermal crystallization and maintain mechanical properties [32]. The activation energy is required to overcome the energy barrier between the disordered liquid phase and the ordered solid phase. Once this energy is supplied, the process of nucleation can occur [33]. In the context of crystallization, activation energy refers to the energy required to initiate the formation of crystalline neat. The activation energy can also affect the crystal growth rate, as a higher activation energy means the reaction will occur slower. An evaluation of the activation energy of a non-isothermal crystallization is critical in materials science and engineering as it can provide valuable information on the nucleation and growth processes of the crystalline phase during cooling. This information can be used to optimize processing conditions and tailor the properties of the composite material. It can also be used for developing customized composite materials with tailored properties for specific applications [34,35].

The differential scanning calorimetry (DSC) technique is commonly used for the kinetic analysis of polymers and for evaluating how material properties change over time and temperature [19]. Applying kinetic methods can yield kinetic parameters (relative crystallinity and activation energy) from DSC measurements [36,37]. There are many methods for analyzing the kinetic data of non-isothermal reactions, and they can be generally divided into model-fitting methods and iso-conversive methods [38,39,40,41]. Model-fitting methods are, for example, the Kissinger and Coats–Redfern (C–R) methods [40,41]; iso-conversive methods can be classified into integral and differential iso-conversive methods [42,43,44]. The most popular iso-conversive integral methods are the Flynn–Wall–Ozawa (FWO) and Kissinger–Akahira–Sunose (KAS) methods. Both methods calculate accurate activation energies [45,46].

The kinetic analysis of PA66 can guide the crystallization process of PA66 in an industrial context. It can also establish a theoretical basis for the development of an action plan and the industrialization of the product. This study complements the previous one [1,2]: Makhlouf and Layachi et al. [1,2] examined the development of crystalline spherulites and crystallization kinetics of PA66/GF composites. Additionally, the optimal loading rate of GF for producing composites tailored for specific applications was revealed.

This study introduces the kinetics and mechanisms of non-isothermal crystallization of a PA66 matrix reinforced with 30 wt.% glass fiber and 0.4 wt.% carbon black. The novelty of the present work is related to (1) the exploration of the kinetic non-isothermal behavior of PA66 composites that are reinforced with both glass fiber and carbon black, through differential scanning calorimetry (DSC), and (2) the determination of the kinetic parameters and activation energy (Ea) and the evaluation of the suitability of the methods proposed. The activation energy of neat PA66, PA66/GF, PA66/CB, and PA66/GF/CB was evaluated using iso-conversive integral methods (Flynn–Wall–Ozawa (FWO) and Kissinger–Akahira–Sunose (KAS)) and model-fitting methods (Kissinger and Coats–Redfern (C–R)). The Criado and Coats–Redfern methods [40,41] were used to identify possible reaction models of neat PA66 and related composites, as well as the nucleating effect of GF and CB on the PA66 matrix, to gain a better understanding of the thermal crystallization mechanism of PA66 and its composites.

## 2. Experimental

### 2.1. Materials

Neat PA66 and composites containing 30 wt.% GF with and without CB were supplied by Solvay (Saint Fons—Belle Etoile, Lyon, France). Four sample types were studied, consisting of PA66 reinforced with 30 wt.% short GF with and without 0.4 wt.% CB (Table 1). The PA66 molecular weight, Mn ≈ 33 kg/mol, was determined by viscosimetry at 30 °C in formic acid, according to the manufacturer. Prior to their use, the glass fibers were surface-treated with an amino-silane coupling agent to improve adhesion between the fibers and the matrix. The effect of coupling agents has been the subject of a number of articles [19,47,48] that have demonstrated the benefits of these agents for improved interfacial adhesion. It should be noted that a concentration of 0.4 wt.% CB is commonly used in mechanical parts.

### 2.2. Scanning Electron Microscopy (SEM)

SEM experiments were performed on a JOEL 840 A LOGS (Tokyo, Japan) apparatus at an acceleration voltage of 1–5 kV. Observations were made from the metal-coated surface of samples broken in liquid nitrogen.

### 2.3. Wide-Angle X-ray Scattering (WAXS)

Experiments involving wide-angle X-ray scattering (WAXS) were conducted at room temperature using a PANalytical Empyrean X-ray diffractometer (Eindhoven, The Netherlands). The diffractometer operated at 40 kV and 28 mA, utilizing the K*α* radiation from a copper anode (λ = 0.154 nm). Data were gathered within the 2θ range of 10° to 35° using a fixed-time mode with a step interval of 0.02°.

### 2.4. Differential Scanning Calorimetry (DSC)

DSC analyses were performed under non-isothermal conditions, using a DSC7 device from Perkin Elmer, based in Waltham, MA, USA, for evaluating the crystallization process under non-isothermal conditions. Samples of about 8 mg, from injection-molded specimens, were first heated up to 290 °C for 5 min to erase their thermal history from the injection-molding process.

The sample was then progressively cooled to 25 °C using variable cooling rates: 2, 5, 10, and 25 °C/min. Throughout the crystallization process, the relative degree of crystallinity was assessed by observing the exothermic reactions of heat released during the cooling phase.

## 3. Results and Discussion

### 3.1. Morphology

**Figure 1:**
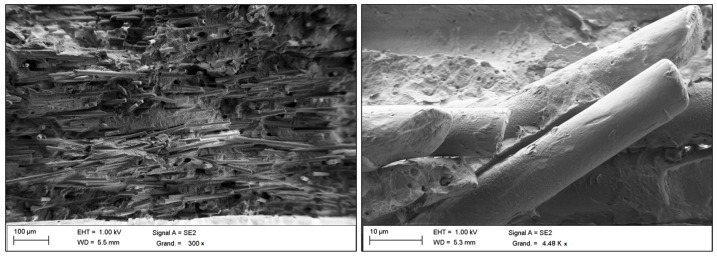
SEM micrographs of a PA66/GF composite.

Figure 1 shows SEM micrographs of the fracture surfaces of (GF)-reinforced PA66 composites treated with a silane-coupling agent. These observations were made to highlight the impact of a coupling agent on the interface between fillers and matrix. In this close-up SEM image, the PA66 matrix is strongly bonded to the GF at the fracture surface. This observation confirms the effective adhesion between the PA66 matrix and the GF, which is due to the aminosilane-based surface treatment applied to the GF.

### 3.2. Microstructural Characterization

**Figure 2:**
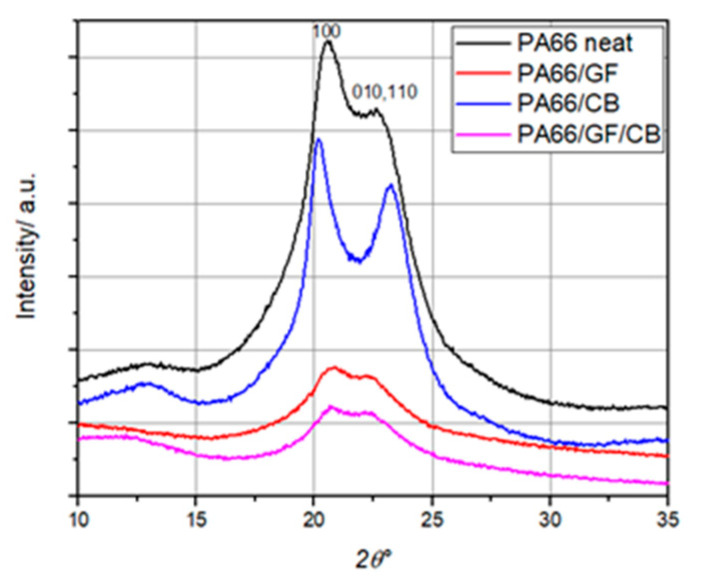
Diffractogram (WAXS) for PA66 and PA66 composites.

The structure of PA66 and composite containing 30 wt.% GF reinforcement, with and without CB, was analyzed using the wide-angle X-ray scattering (WAXS) technique. Collected data, represented as diffractograms (Figure 2), confirmed the presence of two diffraction peaks: the (100) peak and the (010/110) doublet. These peaks correspond to the *α*-triclinic crystalline phase of PA66 [49,50], with scattering angles of 2*θ* = 20° and 2*θ* = 23°, respectively. Diffractogram measurements were conducted over a range of 2θ angles from 10° to 35°. To assess crystallite size, Scherrer’s formula was applied [51].

(1) $$L=\frac{0.9\;\lambda }{2∆\theta \cos\theta }$$

where λ is the wavelength (0.154 nm for the copper K*α* line), Δ*θ* is the width at half-height of the diffraction peak, and *θ* is the angle of the diffraction peak.

Thus, using Scherrer’s expression (1), we estimated the sizes of the crystal diffraction peaks, namely, L(100) and the doublet L(010/110), which were associated with the *α*-crystalline phase of PA66. Table 2 summarizes crystal dimensions, including values for pure PA66 and some composites. The obtained results indicate that reinforcements such as GF and CB lead to a reduction in crystal size. This feature aims to analyze the impact of these reinforcements on crystallite dimensions.

### 3.3. Non-Isothermal Crystallization Behavior

**Figure 3:**
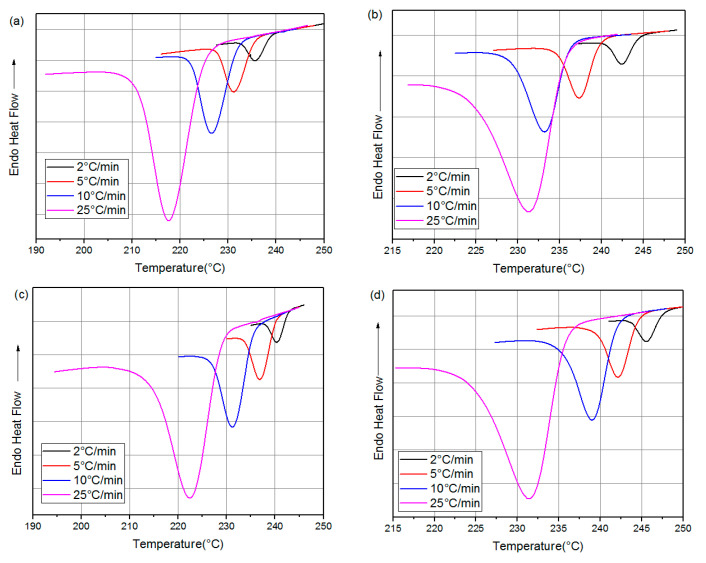
Non-isothermal crystallization exotherms for different cooling rates: (**a**) neat PA66, (**b**) PA66/GF, (**c**) PA66/CB, and (**d**) PA66/GF/CB.

Crystallization curves for both neat PA66 and its composites are shown in Figure 3. It is clear that the crystallization parameters, namely, crystallization temperature (T_c_) and onset crystallization (T_onset_), show a shift towards higher temperatures with the addition of GF and CB [1]. The increase in T_onset_ after the addition of GF and CB suggests that these reinforcing materials promote PA66 crystallization [2,25,29]. Furthermore, the width of the crystallization curve at half maximum is influenced by the cooling rate: the greater the width, the wider the peak. This increase in the amplitude of the crystallization peak, combined with the rise in Tc, indicates that in the presence of GF and CB, the crystallization of PA66 chains occurs more quickly [2,25]. Consequently, it is plausible that the incorporation of reinforcements such as glass fibers not only accelerates the initial formation of crystallization nuclei but also speeds up the overall crystallization process [1,2,25].

The crystallinity rate of the different samples was determined by comparing their melting enthalpy of perfectly crystalline PA66 in the α-crystal form [52].

(2) $$X_{C}\left(\%\right)=\frac{{∆H}_{f}}{{∆H}_{f}^{0}}$$

where ΔH_f_ is the melting enthalpy of PA66 in the samples and ΔH_f_^0^ = 197 j.g^−1^ is the melting enthalpy of perfectly crystalline PA66 in the *α*-crystal form.

Table 3 shows the DSC parameters and results of samples at different cooling rates (β) such as T onset, Tc, enthalpy of fusion ΔH_f_, and the crystallinity ratio X_c_ (%). The data presented in Table 3 clearly show that the exothermic curves of all the samples examined follow a single-peak pattern, considering the cooling rate applied. As the cooling rate increases, there is a shift in the crystallization T_onset_ towards lower temperatures, as indicated by [25,53]. Moreover, this increase leads to a reduction in the crystallization peak temperature Tc for all samples as a whole. In their work, Layachi et al. [1] observed that the crystallization rate at low temperatures is faster than at high temperatures.

The following Equation (3) calculates the relative crystallinity (X(T)) of the polymer matrix as a function of temperature at various cooling rates under non-isothermal conditions [25,54].

(3) $$X\left(T\right)=\frac{\int_{T_{0}}^{T}\left(\frac{{dH}_{C}}{dT}\right)dT}{\int_{T_{0}}^{T_{\infty }}\left(\frac{{dH}_{C}}{dT}\right)dT}=\frac{B_{0}}{B_{\infty }}$$

where B_0_ and B_∞_ are the areas of the DSC cooling scans from T_0_ to T and from T_0_ to T_∞_, respectively, T_0_ and T_∞_ denote the beginning (onset) and ending (offset) of the crystallization peak, and (dHc/dT) is the heat flow during crystallization.

**Figure 4:**
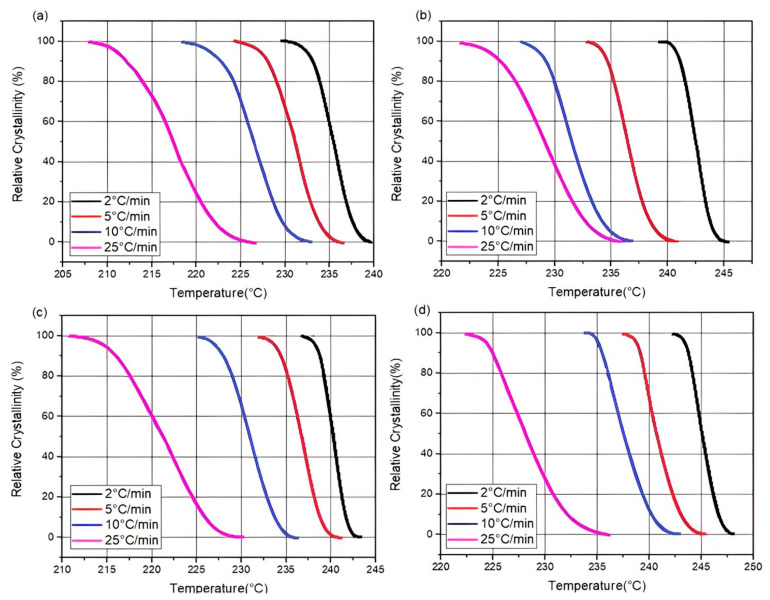
Relative crystallinity X(T) as a function of temperature for different cooling rates: (**a**) neat PA66, (**b**) PA66/GF, (**c**) PA66/CB, and (**d**) PA66/GF/CB.

Figure 4 shows the variation in relative crystalline linearity X(T) of PA66 and its composites at different cooling rates. Transformation ratios, also known as relative crystallinity, frequently follow sigmoidal (“S”) curves. During transformation, the rate of progression remains low, peaking during an intermediate phase before regressing to lower values [1,2,25]. This initial reduction in rate can be explained by the time required to form a significant number of crystallization nuclei. This is before crystal growth begins. The intermediate phase is maintained at a constant level and then accelerates as crystal growth, dependent on the base phase, predominates over germination. At the end of the transformation, the amount of untransformed material decreases considerably. This has a direct impact on the slow formation of new crystallization nuclei. Existing particles come into contact, resulting in a boundary where growth stops [28,55]. Figure 4 clearly shows that sigmoidal shapes are shifted towards lower temperatures as the cooling rate increases, accelerating crystallization. Clearly, the cooling rate is important for the crystallization process. As for the crystallization ratio, it is higher for all composites than for virgin PA66. This allows us to conclude that the GF fillers act as a nucleating agent and thus accelerate the overall crystallization process of the PA66 matrix [22,25].

### 3.4. Kinetics of Non-Isothermal Crystallization

To further investigate the non-isothermal crystallization behavior of the different materials, model-free and model-fitting approaches were also used. Kinetic analysis frequently uses these techniques. The research on non-isothermal crystallization kinetics is best suited to Friedman’s technique [56,57,58]. The kinetic equation of this approach can be used to characterize the crystallization response for all varieties of non-isothermal kinetics investigations [59]. The kinetic equation of this approach can be expressed as follows [58,59]:

(4) $$\frac{dX(T)}{dt}=K\left(T\right)f\left[X\left(T\right)\right]$$

where K(T) is the Arrhenius constant rate, X(T) is relative crystallinity, and f[X(T)] is the function representing the reaction mechanism. In Formula (4), the constant rate K(T) complies with the Arrhenius equation [59,60]

(5) $$K\left(T\right)=Aexp\left[\frac{{-E}_{a}}{RT}\right]$$

where A is the pre-exponential factor, Ea is the activation energy, R is the universal gas constant, and T is the absolute temperature. For the kinetic analysis of the non-isothermal data obtained from the DSC experiments, the constant cooling rate (β) is defined as follows [59,60]: $\beta =\frac{dT}{dt}$.

The value of K(T) and (β) may be substituted in Equation (4) to rewrite Equation (6)

(6) $$\frac{dX(T)}{dT}=\frac{A}{\beta }exp\left[\frac{{-E}_{a}}{RT}\right]f\left[X\left(T\right)\right]$$

Equation (6) gives the analytical approaches’ crystallization rate expression for estimating the (Ea) using DSC kinetics data. Different methods for calculating the activation energy can be used to better understand the crystallization behavior of PA66 and its composites [58,59].

The activation energy can be calculated using different techniques. Activation energies can be predicted by iso-conversional approaches using a model that is independent of various cooling rates. Model-free methods include Kissinger–Akahira–Sunose (KAS) [45,61] and Flynn–Wall–Ozawa (FWO) [46].

#### 3.4.1. Flynn–Wall–Ozawa (FWO) Method

One of the most popular linear integral iso-conversional methods for determining activation energy at various cooling rates is the FWO approach. In this procedure [46,62], trials are conducted at various cooling rates and temperatures corresponding to fixed values of relative crystallinity are estimated as follows [63]:

(7) $$\ln\left(\beta \right)=\left[ln\left(\frac{AE}{g\left[X\left(T\right)\right]R}\right)-5.331\right]-\frac{1.052E_{a}}{RT}$$

where g[X(T)] is the integral kinetic function or integral reaction model.

The slope of the graphs ln(β) versus 1/T gives the activation energy values.

#### 3.4.2. Kissinger–Akahira–Sunose Method

An updated method was proposed by Akahira et al., and it offers a considerable improvement in activation energy values [61]. The empirical estimation of the Arrhenius integral serves as the foundation for the KAS approach [45,64]. It is mostly utilized for composite material calculations of the apparent activation energy at various cooling rate values.

(8) $$\ln\left(\frac{\beta }{T^{2}}\right)=ln\left(\frac{AR}{g\left[X\left(T\right)\right]E_{a}}\right)-\frac{E_{a}}{RT}$$

where Ea, g[X(T)], A, and R refer to the activation energy, integral conversion function, pre-exponential factor, and universal gas constant, respectively. Similarly, using graphs ln ($\frac{\beta }{T^{2}}$) versus (1/T) should thus result in straight lines, and the activation energy may be determined from the slope (Ea/R).

**Figure 5:**
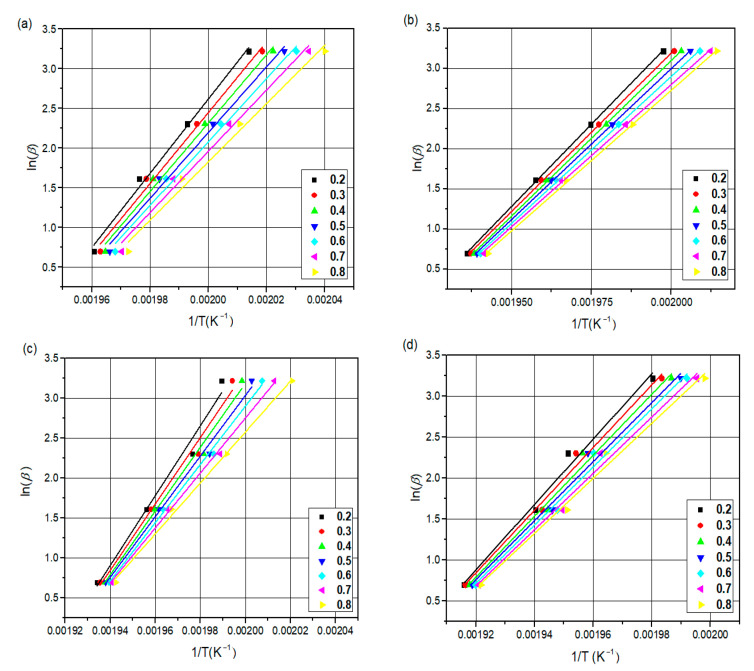
FWO plots at different X(T): (**a**) neat PA66, (**b**) PA66/GF, (**c**) PA66/CB, and (**d**) PA66/GF/CB.

**Figure 6:**
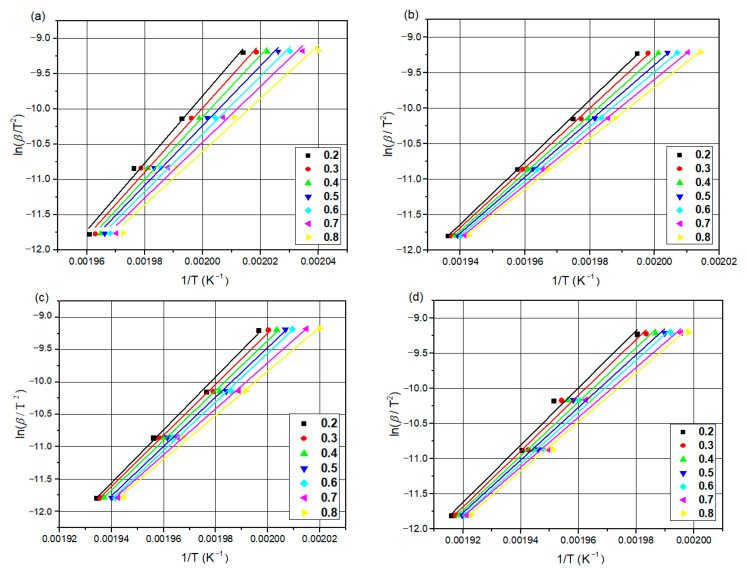
KAS plots at different X(T): (**a**) neat PA66, (**b**) PA66/GF, (**c**) PA66/CB, and (**d**) PA66/GF/CB.

The activation energy of the non-isothermal crystallization of neat PA66, PA66/GF, PA66/CB, and PA66/GF/CB composites was calculated using FWO and KAS methods. Figure 5 and Figure 6 show the FWO and KAS plots for different X(T) values of neat PA66, PA66/GF, PA66/CB, and PA66/GF/CB.

The R^2^ values from FWO and KAS are 0.9945 and 0.9756, respectively (Figure 5 and Figure 6). This result demonstrates that both methods may be used to investigate the crystallization kinetics of these systems.

The activation energy of neat PA66 is greater than that of PA66/CB and PA66/GF in the X(T) range of 0.2 to 0.8. The addition of GF and CB to the PA66 matrix reduces the Ea from 280.08 to 314.27 KJ/mol by both FWO and KAS methods. The presence of GF and CB changes the crystallization rate of PA66 [1]; the addition of GF and CB in the PA66 matrix leads to an increase in the crystallization rate of PA66 composites because activation energy is a barrier that must be overcome before a chemical reaction can occur, and a lower activation energy value means that an easier reaction can occur [65,66,67].

Table 4 and Table 5 show the relationship of Ea as a function of X(T) obtained by these iso-conversional methods (FWO and KAS).

**Figure 7:**
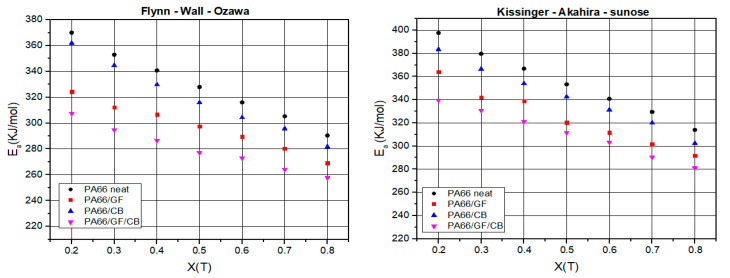
Variations of activation energy values as a function of X(T) of neat PA66, PA66/GF, PA66/CB, and PA66/GF/CB obtained from the FWO and KAS methods.

Figure 7 graphically shows the plots of Ea against X(T) of neat PA66, PA66/GF, PA66/CB, and PA66/GF/CB. It is clear that Ea values decrease with X(T) ranging from 0.2 to 0.8 in a linear way, which confirms that it has similar types of entity formation crystals during the crystallization process [1]. This behavior reveals a single-process reaction [63,65], with the FWO method showing lower values than the KAS method.

#### 3.4.3. Kissinger Method

**Figure 8:**
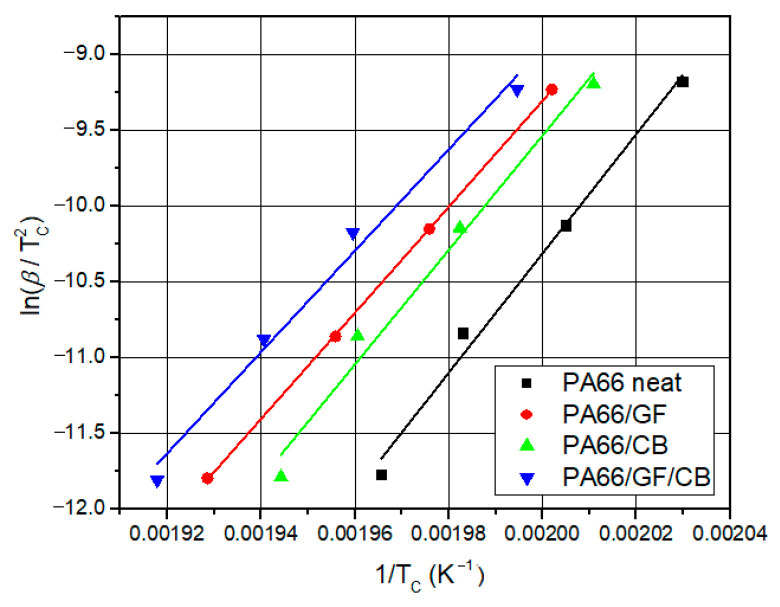
Kissinger fitting plots of neat PA66, PA66/GF, PA66/CB, and PA66/GF/CB.

The activation energy for non-isothermal crystallization can be derived from the combination of peak crystallization temperature and cooling rate [1]. The only parameter that can be estimated using model-free techniques is the activation energy, whereas the activation energy and the reaction mechanism can both be computed using the model-fitting method [68]. Other kinetic characteristics can also be derived, including the best-fit reaction mechanism function and the pre-exponential factor. To determine the activation energy for non-isothermal crystallization, Kissinger [69] provided an equation. With this approach, calculating the Ea for every value of X(T) is not essential. The equation provides the activation energy [61,69].

(9) $$\ln\left(\frac{\beta }{T_{C}^{2}}\right)=ln\left(\frac{AR}{E_{a}}\right)+ln\left(-\frac{df[X\left(T\right)]}{dX(T)}\right)-\frac{E_{a}}{RT_{C}}$$

where β is the cooling rate, R is the universal constant of perfect gases (8.314 J K^−1^ mol^−1^), Tc is the peak crystallization temperature, and Ea is the energy of activation at different cooling rates. The activation energy is estimated from the slope of the curve ($ln\beta /{Tc}^{2}$) as a function of 1/T_C_ (Figure 8). The activation energy values for the non-isothermal crystallization of neat PA66, as well as the different composites, are shown in Table 6. In this approach, the Ea does not provide any information on the variance of the activation energy during thermal crystallization; instead, it merely displays the median value of the activation energy, or “apparent energy”, throughout the whole process [36]. Thus, the FWO and KAS procedures are also applied in this situation. The Ea values calculated using the Kissinger approach and the FWO and KAS methods for PA66 and its composites are in good agreement. The Ea for neat PA66 using the Kissinger technique is 327.83 KJ/mol, which compares to the 310 KJ/mol results calculated by Layachi et al. [1]. The activation energy is reduced by the addition of GF by a small amount, 291.26 KJ/mol.

### 3.5. Reaction Order and Mechanism

The kinetic study provides a mathematical and theoretical explanation for what occurs empirically [70,71,72]. In solid-state reactions, a model can describe a particular type of reaction and theoretically convert it into a formula. Many models rely on certain mechanical considerations [70]. The Coats–Redfern and Criado approaches are used since they incorporate the crystallization process to determine the kinetic model for the non-isothermal crystallization of neat PA66 and its composites.

#### 3.5.1. Coats–Redfern (C–R) Method

C–R equations have the following form [73]:

(10) $$Y=ln\left[\frac{1-{\left(1-X\left(T\right)\right)}^{1-n}}{(1-n)T^{2}}\right]=ln\left[\frac{AR}{\beta E_{a}}\left(1-\frac{2RT}{E_{a}}\right)\right]-\frac{E_{a}}{RT}\;for\;n\neq 1$$

(11) $$Y=ln\left[\frac{-ln(1-X\left(T\right))}{T^{2}}\right]=ln\left[\frac{AR}{\beta E_{a}}\left(1-\frac{2RT}{E_{a}}\right)\right]-\frac{E_{a}}{RT}\;for\;n=1$$

where *n* is the reaction order and β the cooling rate.

The kinetic triplet is calculated using this approach (Ea, A, and reaction order *n*) [73]. By graphing $ln\left[\frac{1-{\left(1-X\left(T\right)\right)}^{1-n}}{(1-n)T^{2}}\right]\;$ versus 1/*T* for Equations (10) and (11), the activation energy and pre-exponential factor can be calculated. Furthermore, 2RT/Ea has an extremely low value, less than 1. Consequently, the entire expression may be thought of as a constant [74]. The values of Ea/R and Ln (AR/β Ea) are determined by the slope and intercept, respectively. In contrast to the FWO and KAS approaches, where the Ea values rely on X(T), the C–R method yields a unique value for Ea. It is interesting that the FWO and KAS approaches are difficult to use to determine the reaction order but are effective for assessing Ea without knowledge of the reaction model (*n*) [59].

**Figure 9:**
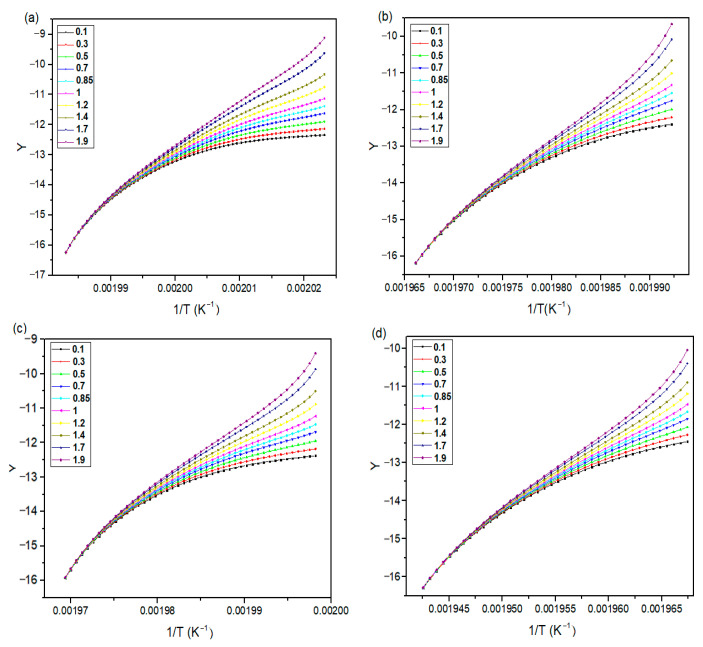
Determination of activation energies using the C–R method for (**a**) neat PA66, (**b**) PA66/GF, (**c**) PA66/CB, and (**d**) PA66/GF/CB at the cooling rate of 10 °C/min.

The selection of (*n*) in the C–R method is verified when the regression coefficient (R^2^) of Formulas (10) and (11) is near 1. Formulas (10) and (11) in this study employ several reaction orders ranging from *n* = 0.1 to 2.0. Plots for neat PA66 and its composites for various (*n*) values at a cooling rate of 10 °C/min are shown in Figure 9. At all cooling rates, the same tendency is seen in all curves. A comparison of Ea for neat PA66 and its blends is shown in Table 7. Moreover, it should be noted that the C–R approach follows the same pattern as the FWO and KAS methods. It is evident that the activation energy values acquired by the C–R method are larger than those obtained by the FWO and KAS methods [59]. This disparity can be explained by the model-fitting methodology, which involves fitting several models to the relative crystallinity curve vs. the temperature curve before calculating the activation energy and pre-exponential factor [75].

#### 3.5.2. Crystallization Mechanism by the C–R Method

The iso-conversional method (FWO and KAS) offers the benefit of determining activation energy for individual alphas, whereas the fitting method is characterized by its minimal data requirements and the correspondence of a single activation energy to an average advancement degree. The fitting method is primarily suitable for simple mechanisms. Additionally, we also utilize the CR method, which is another fitting technique. Its advantage lies in the ability to calculate a mechanism degree and model-fitting [73]. The integral version of the C–R technique in Equation (12) is frequently presented as follows:

(12) $$ln\left(\frac{g\left[X\left(T\right)\right]}{T^{2}}\right)=-\frac{E_{a}}{R}\cdot \frac{1}{T}+ln\frac{AR\cdot \left(1-\frac{2RT}{E_{a}}\right)}{\beta E_{a}}$$

**Figure 10:**
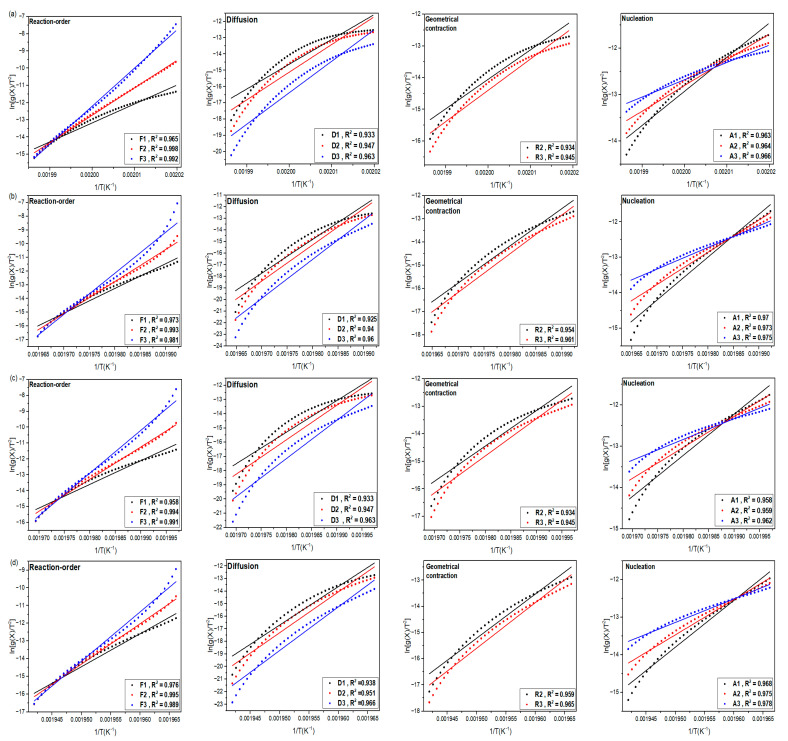
Fitted results according to the integral form of Coats–Redfern method for (**a**) neat PA66, (**b**) PA66/GF, (**c**) PA66/CB, and (**d**) PA66/GF/CB at the cooling rate of 10 °C/min.

Table 8 lists the theoretical functions of f[X(T)] and g[X(T)] [33]. Based on the C–R equation [76], the straight lines ought to be given by the linearity fitting of the logarithmic term of g[X(T)] over 1/T. The most likely function should be determined by the best regression coefficient (R^2^). According to the C–R approach, Figure 10 displays the fitted plots using various theoretical functions. With (R^2^) equal to 0.998, only the F2 kinetic models exhibit high straight-fitting levels. According to the F2 reaction model, each particle randomly has two nuclei throughout the nucleation process [77]. In this process, nuclei form at sample flaws caused by contaminants and edges inside the sample [33].

### 3.6. Confirmation of Crystallization Mechanism by Criado Method

Selecting the appropriate kinetic model can be challenging in cases where the (R^2^) of kinetic models produced using the integral version of the C–R approach is higher than 0.99. The Criado approach was employed in this work to validate the appropriate kinetic model and to establish the kinetic models [76]. Equation (13) calculates experimental values as follows:

(13) $$Z\left[X\left(T\right)\right]=\frac{E_{a}}{R}\frac{dX\left(T\right)}{dT}e^{\frac{E_{a}}{RT}}P\left(x\right)$$

where P(x) can be expressed by Equation (14) [59,79,80], with a minimal error level of 10^−5^% for x > 20:

(14) $$P\left(x\right)=\frac{e^{-x}}{x}\frac{x^{3}+18x^{2}+86x+96}{x^{4}+20x^{3}+120x^{2}+240x+120}$$

where x is Ea/RT.

The theoretical graphs were generated by plotting Z(X(T)) against X(T) for different reaction mechanisms using Equation (15) and utilizing the g (X(T)) and f(X(T)) values from Table 8. The resulting functions were categorized into reaction order, diffusion, geometrical contraction, and nucleation based on the underlying assumptions of the respective mechanisms [81]:

(15) $${Z[X\left(T\right)]}_{theorical}=g\left[X\left(T\right)\right]\cdot f\left[X\left(T\right)\right]$$

**Figure 11:**
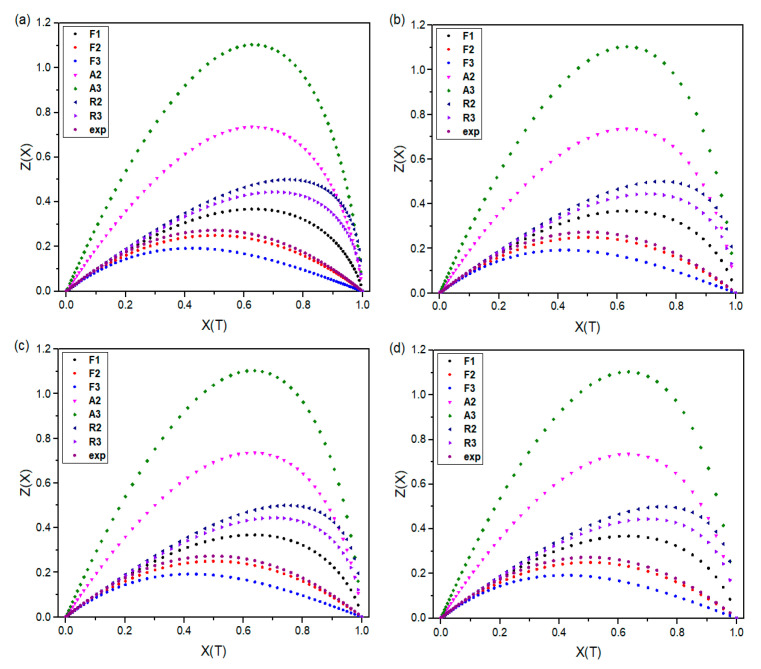
Reaction mechanism plots using the Criado method for (**a**) neat PA66, (**b**) PA66/GF, (**c**) PA66/CB, and (**d**) PA66/GF/CB at the cooling rate of 10 °C/min.

The proper non-isotherm crystallization process was then determined by comparing the experimental curves with several theoretical plots. The master curves of PA66 and its composites were plotted using Ea and A determined by the C–R technique to demonstrate the non-isotherm crystallization process [80,81]. Figure 11 displays generated Z(X(T)) graphs. When compared to the master plots, this experimental plot offers a quick and precise evaluation of the crystallization mechanism. In this study, the kinetic model was confirmed by Coats–Redfern and Criod methods. The non-isotherm crystallization for neat PA66, PA66/GF, PA66/CB, and PA66/GF/CB follows the second-order reaction (F2) with the integral form g(X(T)) = [(1 − X)^−1^] − 1 and the differential form f(X(T)) = (1 − X)^2^. The findings provide useful insights for understanding the non-isotherm crystallization mechanism of neat PA66 and its composites.

## 4. Conclusions

This paper investigates the effect of GF on PA66 crystallization behavior. The addition of a small amount of carbon black was investigated, owing to its widespread occurrence in industrial parts. Thermal crystallization kinetics of neat PA66 and its composites were analyzed using model-free and model-fitting approaches. The thermal crystallization results of the composites compared to neat PA66 show that GF promotes a nucleation effect. This significantly increases the crystallization kinetics and crystallinity ratio of the PA66 matrix in the composites. The crystallization rate also increased with the addition of CB despite the rather low content. The experimental results indicate that the presence of GF and CB improves the thermal stability of PA66 composites. Moreover, the average energy of neat PA66, as determined by the FWO method, stands at 329.1 KJ/mol, surpassing the energies of PA66/GF, PA66/CB, and PA66/GF/CB, which measure 297.1, 319.7, and 280.1 KJ/mol, respectively. It is clear from this that GF and CB contribute to the crystallization of PA66 in a synergistic manner. Crystallization of neat PA66, PA66/GF, PA66/CB, and PA66/GF/CB follows the second-order (F2) with the integral form g(X(T)) = $\left[{\left(1-x\right)}^{-1}\right]-1$ and the differential form f(X(T) = (1−x)², i.e., the reactions are decelerated, showing that PA66 matrix presents good affinity with GF and CB fillers. The results provide useful insights into the crystallization mechanism of pure PA66 and its composites.

## Figures and Tables

**Table 1:** Samples.

Samples	PA66 (%)	GF (%)	CB (%)
Neat PA66	100	0	0
PA66/GF	70	30	0
PA66/CB	99.6	0	0.4
PA66/GF/CB	69.6	30	0.4

**Table 2:** Crystal size of PA66 and PA66 composites.

Samples	L100	L010/110
Neat PA66	3.99	3.82
PA66/GF	3.95	5.06
PA66/CB	7.07	2.95
PA66/GF/CB	3.33	5.99

**Table 3:** DSC parameters of samples at various cooling rates (β).

Samples	(β) (°C/min)	T_onset_ (°C)	Tc (°C)	T_offset_ (°C)	ΔH_f_ (j/g)	X_c_ (%)
Neat PA66	2	239.2 ± 0.3	235.6 ± 0.4	231.8 ± 0.3	75.2 ± 0.4	38.1 ± 0.2
	5	236 ± 0.6	231.1 ± 0.4	226 ± 0.6	71.1 ± 0.5	36.1 ± 0.4
	10	232 ± 0.8	226.6 ± 0.6	220.3 ± 0.5	68.3 ± 0.7	34.6 ± 0.3
	25	225.7 ± 1	217.8 ± 0.7	208.2 ± 0.7	67.1 ± 0.8	34 ± 0.6
PA66/GF	2	244.7 ± 0.5	242.4 ± 0.6	240.4 ± 0.4	99.5 ± 0.4	50.5 ± 0.3
	5	240 ± 0.5	237.3 ± 0.5	234.2 ± 0.7	99 ± 0.8	50.2 ± 0.3
	10	236.5 ± 0.6	233 ± 0.7	228.3 ± 0.6	86.8 ± 0.9	44.1 ± 0.4
	25	235.7 ± 0.9	231.4 ± 0.7	222 ± 0.8	81.7 ± 1	41.4 ± 0.2
PA66/CB	2	242.6 ± 0.4	240.4 ± 0.3	238 ± 0.6	76 ± 0.7	38.6 ± 0.1
	5	240.3 ± 0.7	237 ± 0.6	233.6 ± 0.6	75.3 ± 0.8	38.2 ± 0.3
	10	235.5 ± 0.9	231.3 ± 0.9	226.8 ± 0.7	73.1 ± 1.1	37.1 ± 0.5
	25	229.5 ± 1	222.4 ± 0.8	211.6 ± 0.9	61.2 ± 0.9	31 ± 0.2
PA66/GF/CB	2	247.5 ± 0.6	245.5 ± 0.6	243.6 ± 0.5	102.7 ± 0.3	52.1 ± 0.4
	5	245 ± 0.8	242 ± 0.6	238.2 ± 0.4	93.8 ± 0.7	47.6 ± 0.6
	10	242.6 ± 0.6	239 ± 0.9	234.6 ± 0.6	92.1 ± 0.8	46.7 ± 0.5
	25	235.7 ± 1.1	231.5 ± 0.9	224.5 ± 1	87.3 ± 1	44.3 ± 0.6

**Table 4:** Variation of activation energies and average activation energies of samples determined from FWO at different X(T).

X(T)	Neat PA66	PA66/GF	PA66/CB	PA66/GF/CB
	−Ea (KJ/mol)			
0.2	370.0	324.4	361.9	307.3
0.3	352.9	312.3	344.7	294.7
0.4	340.7	306.6	323.8	286.5
0.5	327.9	297.5	315.9	277.1
0.6	316	289.5	304.3	273
0.7	305.3	280.3	295.7	264.1
0.8	290.5	269.2	291.8	257.7
average	329.1	297.1	319.7	280.1

**Table 5:** Variation of activation energies and average activation energies of samples determined from KAS at different *X*(*T*).

*X(T)*	Neat PA66	PA66/GF	PA66/CB	PA66/GF/CB
	−Ea (KJ/mol)			
0.2	397.6	364.1	383.5	339.5
0.3	379.7	342	366.6	330.8
0.4	366.8	338.8	359	321. 1
0.5	353.3	320.4	347.6	311.6
0.6	340.8	311.7	331.3	307.4
0.7	329.5	301.6	320	298.4
0.8	313.8	291.8	310.4	291
average	354.5	324.3	345.5	314.3

**Table 6:** Apparent activation energies (R^2^) of samples determined by the Kissinger method.

Samples	−Ea KJ/mol	R^2^
Neat PA66	327.8	0.97223
PA66/GF	291.2	0.99536
PA66/CB	317. 9	0.97734
PA66/GF/CB	283.2	0.99328

**Table 7:** Activation energies, average activation energies, and reaction orders (*n*) of samples determined by the C–R method at various cooling rates.

Samples	2 °C/min		5 °C/min		10 °C/min		25 °C/min		Ea_Average_
	Ea	*n*	Ea	*n*	Ea	*n*	Ea	*n*	
Neat PA66	994.7	1.7	676.9	1.4	666.5	1.9	423.6	1.7	690.4
PA66/GF	836.3	1.7	556.0	1.9	404.7	1.9	362.4	1.9	539.8
PA66/CB	915.7	1.7	664.5	1.4	649.8	1.9	415.3	1.7	661.3
PA66/GF/CB	740.3	1.7	517.5	1.7	394.6	1.9	312.3	1.9	491.2

**Table 8:** Algebraic expressions of gX(T) and fX(T) for the most common reaction mechanisms of the solid-state process [33,59,78].

Mechanism	Solid State Process	f[X(T)]	g[X(T)]
Diffusion Dn	One-dimensional diffusion D1	$\frac{1}{2\cdot X}$	$X^{2}$
	Two-dimensional diffusion (Valensi model) D2	${\left[-ln(1-X)\right]}^{-1}$	$\left(1-X\right)ln\left(1-X\right)+X$
	Three dimensioal diffusion (Jander model) D3	$\frac{3}{2}{\left(1-X\right)}^{\frac{2}{3}}{\left[1-{\left(1-X\right)}^{\frac{1}{3}}\right]}^{-1}$	${\left[1-{\left(1-X\right)}^{\frac{1}{3}}\right]}^{2}$
Reaction order Fn	First order F1	(1−X)	−ln⁡(1−X)
	Second order F2	${\left(1-X\right)}^{2}$	$[{\left(1-X\right)}^{-1}]-1$
	Third order F3	${\left(1-X\right)}^{3}$	$\left[{\left(1-X\right)}^{-2}-1\right]/2$
Avrami–Erofe’ve An Nucleation	Avrami–Erofe’ve A1	$\frac{1}{2}\left(1-X\right)[-ln(1-X{)]}^{\frac{1}{3}}$	${\left[-ln(1-X)\right]}^{\frac{2}{3}}$
	Avrami–Erofe’ve A2	$2\left(1-X\right)[-ln(1-X{)]}^{\frac{1}{2}}$	${\left[-ln(1-X)\right]}^{\frac{1}{2}}$
	Avrami–Erofe’ve A3	$3\left(1-X\right)[-ln(1-X{)]}^{\frac{2}{3}}$	${\left[-ln(1-X)\right]}^{\frac{1}{3}}$
Contraction reaction Rn	Contraction sphere R2	$2{\left(1-X\right)}^{\frac{1}{2}}$	$1-{\left(1-X\right)}^{\frac{1}{2}}$
	Contraction cylinder R3	$3{\left(1-X\right)}^{\frac{2}{3}}$	$1-{\left(1-X\right)}^{\frac{1}{3}}$

## Data Availability

Some or all data, models, or codes that support the findings of this study are available from the corresponding author upon reasonable request.
